# Highly Efficient *Agrobacterium tumefaciens* Mediated Transformation of Oil Palm Using an *EPSPS*-Glyphosate Selection System

**DOI:** 10.3390/plants13233343

**Published:** 2024-11-28

**Authors:** Chao Ouyang, Xiongxia Jin, Qinghui Guo, Shaojie Luo, Yusheng Zheng, Jixin Zou, Baoguang An, Dongdong Li

**Affiliations:** 1 National Key Laboratory for Tropical Crop Breeding, College of Tropical Agriculture and Forestry, Hainan University, Sanya 572025, China; oycsuper@163.com (C.O.); cmxwgg1@163.com (Q.G.); zourich@163.com (J.Z.); 2 College of Tropical Agriculture and Forestry, Hainan University, Haikou 570228, China; jinxiongxiajxx@163.com; 3 School of Life and Health Sciences, Hainan University, Haikou 570228, China; biuryd8927@163.com (S.L.); yusheng.zheng@hainanu.edu.cn (Y.Z.); 4 Coconut Research Institute, Chinese Academy of Tropical Agricultural Sciences, Wenchang 571339, China; 5 Guangdong Bolian Biotechnology Co., Ltd., Guangzhou 511466, China

**Keywords:** oil palm, genetic transformation, embryonic callus, *EPSPS*, glyphosate, *Agrobacterium*-mediated, *mScarlet-Ⅰ*

## Abstract

Oil palm (*Elaeis guineensis* Jacq.) is the most efficient oil-producing crop globally, yet progress in its research has been hampered by the lack of effective genetic transformation systems. The *EPSPS* gene, encoding 5-enolpyruvylshikimate-3-phosphate synthase, has been used as a transgenic selection marker in various crops, including rice and soybean. This study evaluated the *EPSPS*/glyphosate selection system for oil palm transformation. We constructed a binary expression vector, pCGlyDESCLI-C, containing the *TIPS-EiEPSPS* selection marker from goosegrass and the *mScarlet-I* red fluorescent reporter gene. This vector was introduced into oil palm embryonic callus (EC) via *Agrobacterium*-mediated transformation. After optimizing the transformation steps, positive calli were obtained, and integration of the foreign gene into the oil palm genome was confirmed through molecular analysis. Notably, the selection efficiency of the *EPSPS*/glyphosate selection system exceeded that of the traditional *hpt*/hygromycin selection system, demonstrating its advantages. Our findings support the effectiveness of the *TIPS-EiEPSPS*/glyphosate selection system for oil palm genetic transformation, marking its first application in this species and offering a valuable tool for advancing research on this economically significant crop.

## 1. Introduction

As a major tropical oil crop, oil palm (*Elaeis guineensis* Jacq.) is one of the four largest woody edible oil plants globally, alongside coconut(*Cocos nucifera* Linn.), Oil tea camellia (*Camellia* spp.), and olive (*Olea europaea* L.) [1,2]. Oil palm stands out as the most efficient oil-producing crop, contributing 33% of the world’s vegetable oil and 45% of edible oil production, while occupying only 5% of the total cultivated area [3]. In 2023, global palm oil production is projected to reach 79.26 million tons, representing 36% of total vegetable oil output, with an additional 4% attributed to palm kernel oil. Palm oil remains the dominant player in the oils and fats market, followed by soybean and rapeseed oils, which together account for 79% of the total market share.

Genetic transformation technology is an important extension of oil palm breeding techniques. Traditional breeding methods are constrained by a long generation cycle of 10 to 15 years [4] and limited genetic diversity due to species incompatibility. In contrast, genetic transformation overcomes these limitations by broadening the gene pool, accelerating the breeding process, and enabling the introduction of high-value traits that are otherwise unattainable through conventional methods. This technology enhances both the competitiveness and sustainability of the oil palm industry [5]. Moreover, genetic transformation plays a crucial role in advancing basic research on oil palm. Although oil palm transformation research began relatively late, in the late 1980 s, significant progress was made during the 1990s [6,7]. Since then, various studies have focused on factors influencing transformation efficiency, including the types of explants and transformation methods. Despite advancements in these areas [8,9], progress remains slow, and the development of an efficient and stable genetic transformation system continues to pose a significant challenge.

Previous research has shown that transient transfection efficiency in oil palm using PEG-mediated protoplast transformation is 4.22%, increasing to 14% with the use of newer DNA microinjection techniques [10,11]. Stable transformation efficiencies, however, remain low, ranging from 1% to 1.5% using particle bombardment [12] and approximately 0.7% to 1% through *Agrobacterium*-mediated transformation [13,14]. Although stable transformation methods have been developed, efficiency remains considerably lower compared to other crops such as rice, corn, and soybean. Thus, there is an urgent need to explore new strategies to improve the genetic transformation efficiency of oil palm.

Previous studies have shown that certain mutated forms of EPSPS, the target enzyme of glyphosate, are unable to bind to glyphosate, thereby conferring resistance to the herbicide [15,16]. The gene encoding glyphosate-insensitive EPSPS, isolated from microorganisms capable of surviving in high concentrations of glyphosate, has served as a key source for developing glyphosate-resistant transgenic crops [17]. Notably, the *EPSPS* gene derived from *Agrobacterium tumefaciens* strain *CP4* [18], the *AM79aroA* gene from *Escherichia coli* [19,20], and the *Isoptericola variabilis EPSPS* gene (*I. variabilis-EPSPS*) [21] have all been identified. Furthermore, several newly discovered glyphosate tolerance genes from bacteria, including *G2-EPSPS* [22,23], *G10-EPSPS* [17], and *G2m-EPSPS* [24], have been employed as selectable marker genes in the development of glyphosate-tolerant crops. Additionally, a naturally mutated form of *EPSPS*, the Thr102Ile/Pro106Ser double mutant (*TIPS-EPSPS*), exhibited high levels of glyphosate resistance in maize (*TIPS-ZmEPSPS*) [25], goosegrass (*Eleusine indica*) (*TIPS-EiEPSPS*) [15], and rice (*TIPS-EiEPSPS*) [26]. The *TIPS-EPSPS* gene has also been utilized as a transgenic selection marker in maize [27] and rice [26].

The *EPSPS*/glyphosate selection system has been successfully employed for transgenic selection in monocotyledonous crops such as rice [26], wheat [18,28], and corn [27], as well as in dicotyledonous crops like soybean [29,30,31], cotton [32], and Brassica napus [33]. Compared to antibiotic selection marker genes, herbicide-resistant genes offer dual advantages: they can function both as selection markers and as target traits for transgenic plants, thus providing added value. In oil palm genetic transformation research, selection markers such as *bar*, *pmi*, *DOG-1*, and *hpt*, all derived from bacterial sources, have been commonly used [8]. However, the heterologous expression of microbial genes in plants raises safety concerns related to genetically modified organisms, driving interest in exploring plant-derived selection genes. To date, no studies have reported the use of the *EPSPS*/glyphosate selection system in oil palm transformation.

Having previously employed the *TIPS-EiEPSPS*/glyphosate selection system in rice, this study marked the first attempt to test whether the double-mutant *TIPS-EiEPSPS*/glyphosate resistance gene could serve as a selection marker in oil palm. After optimizing conditions at each step, we successfully established this system in oil palm. Importantly, the selection efficiency was significantly higher than that of the traditional *hpt*/hygromycin selection system, demonstrating its clear superiority. Our study offers a promising alternative for advancing both basic and applied research in this crop.

## 2. Results

### 2.1. Determination of the Minimum Inhibitory Concentration of Glyphosate

**Figure 1:**
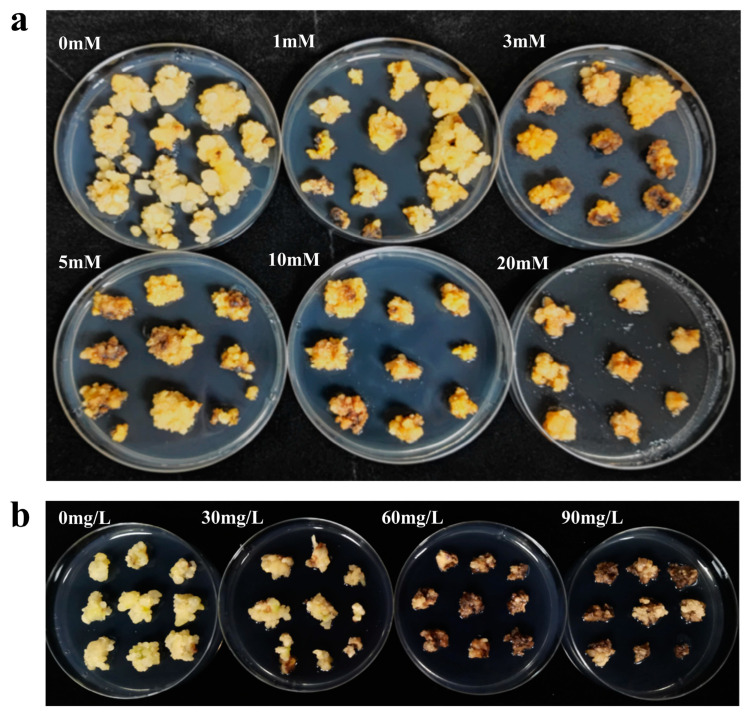
The minimum inhibitory concentration of selection agents tests for the oil palm wild type callus: (**a**) Glyphosate tolerance tests for the oil palm wild type EC; (**b**) hygromycin tolerance tests for the oil palm wild type EC. The numbers represent different concentrations of selection agents adding in the selection medium.

Wild-type embryonic calli (ECs) of oil palm were propagated and placed on selection media containing various concentrations of glyphosate and hygromycin (Table 1). After four to five selection cycles, the growth responses of the ECs varied depending on the concentration of glyphosate and hygromycin applied. The growth status after 13 weeks of selection is depicted in Figure 1a. The callus cultured in a glyphosate-free selection medium exhibited rapid and robust growth, with a new callus formation ratio of 95.56% ± 3.85% (Table 1, Supplementary Table S2). However, in the presence of 1 mM of glyphosate, callus growth was partially inhibited; some exhibited browning and growth inhibition, while others continued to produce new callus, resulting in a reduced new callus formation ratio of 33.33% ± 4.44% (Table 1, Supplementary Table S2). At 3 mM and 5 mM of glyphosate, most callus experienced significant growth inhibition. Although some individual calli initially enlarged, they gradually browned and perished after 16 weeks of selection, with no new callus formation observed. Concentrations above 5 mM of glyphosate severely inhibited growth, with more pronounced browning, and all calli died within 15 weeks. Compared to hygromycin, glyphosate induced a slower rate of browning and had a less immediate effect on callus growth (Figure 1b, Table 1, Supplementary Table S2); however, it ultimately inhibited the size and vitality of non-resistant callus, leading to their death over time. Based on the results of three independent trials, 3 mM of glyphosate was determined to be the minimum inhibitory concentration suitable for use in oil palm genetic transformation. In this study, the *TIPS-EiEPSPS*/glyphosate system was chosen as a novel selection system for oil palm genetic transformation, with the *hpt*/hygromycin system used as a control for comparison in subsequent transformation efficiency evaluations.

### 2.2. Construction of the pCGlyDESCLⅠ-C for Oil Palm Transformation

**Figure 2:**
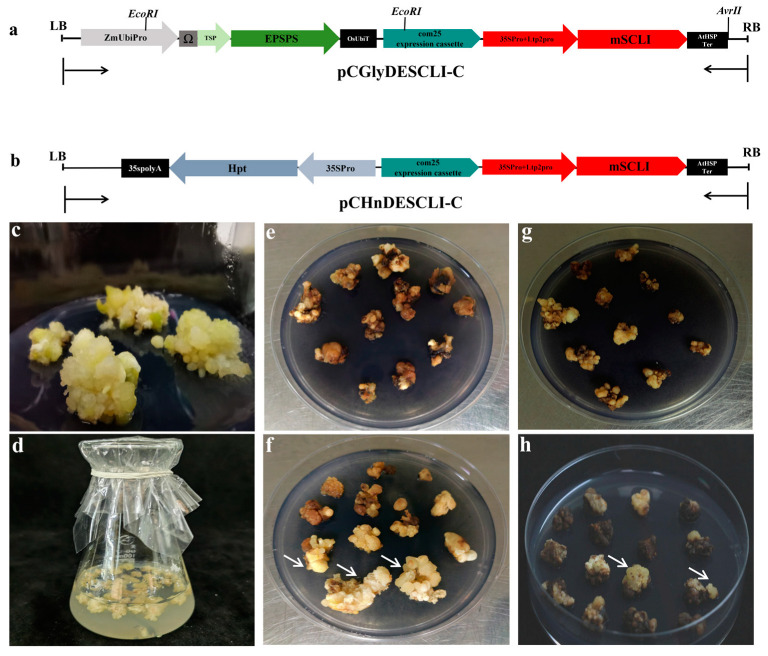
*Agrobacterium*-mediated transformation of oil palm EC using glyphosate based on pCGlyDESCLI-C vector and hygromycin based on pCHnDESCLⅠC: (**a**,**b**) The T-DNA of pCGlyDESCLI-C vector and pCHnDESCLⅠ-C, respectively (Gly, glyphosate; Hn, hygromycin; ZmUbipro, maize ubiquitin promoter; Ω, enhancer; TSP, chloroplast target peptide from tobacco; OsUbiT, rice ubiquitin terminator; 35SPro, CaMV35S constitutive promoter; Ltp2pro, seed-specific promoter; mSCLⅠ, mScarlet-Ⅰ; AtHSP-Ter, heat shock protein terminator of *Arabidopsis thaliana*; Hpt, hygromycin phosphotransferase gene; 35spolyA, CaMV35S polyA terminator; com25,a fatty acid functional gene); (**c**) WT EC; (**d**) infection; (**e**) Wild-type EC on the selection medium containing 3 mM of glyphosate after about 15 weeks of infection; (**f**) Positive calli on the selection medium containing 3 mM of glyphosate after about 15 weeks of infection. (**g**) Wild-type EC on the selection medium containing 60 mg/L of hygromycin after about 15 weeks of infection; (**h**) Positive calli on the selection medium containing 60 mg/L of hygromycin after about 15 weeks of infection. The white arrow heads indicated the suspected resistant calli in (**f**,**h**).

In prior research, we introduced the plant binary expression vector pCEiEPSPS, which contains the *TIPS-EiEPSPS* selection gene, into rice via *Agrobacterium*-mediated transformation, establishing a glyphosate selection system in this species [26]. Given that oil palm is also a monocotyledonous plant, it may share certain characteristics with rice. To facilitate the observation of transformation, we incorporated the *mScarlet-I* (abbreviated as *mSCLⅠ*) reporter, a monomeric protein known for its bright red fluorescence [34]. The resulting expression vector was designated pCGlyDESCLI-C (Figure 2a), with ‘Gly’ indicating glyphosate selection. The pCHnDESCLⅠ-C vector, which differs only in the selection marker gene (hygromycin phosphotransferase) (Figure 2b), served as a control for the pCGlyDESCLI-C vector under identical conditions. Both vectors were transformed into *Agrobacterium* (EHA105) after verification by sequencing.

### 2.3. Optimization of Agrobacterium-Mediated Genetic Transformation

**Figure 3:**
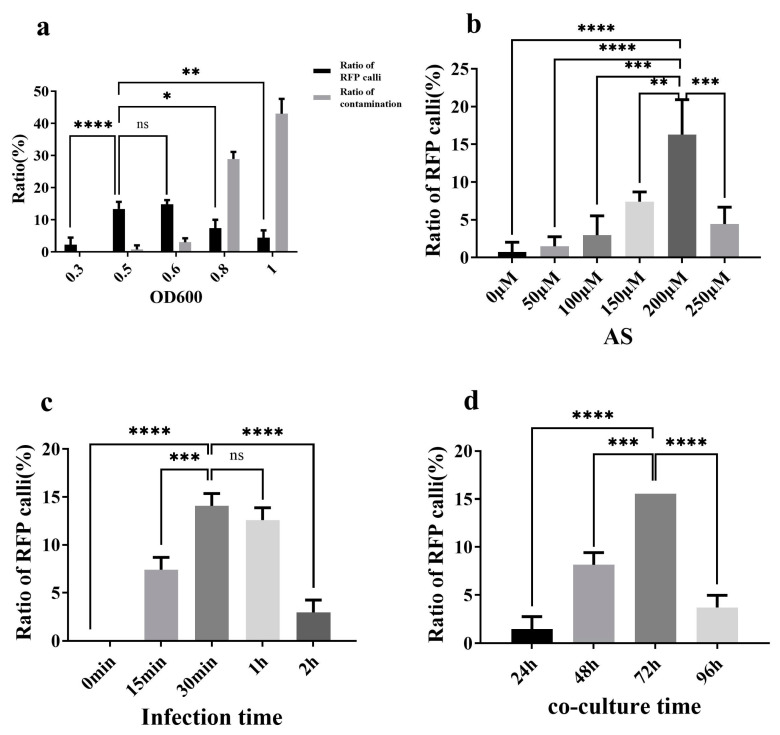
Effects of *Agrobacerium tumefaciens* cell density (OD_600_): (**a**), Acetosyringone (AS) concentration (**b**), infection time (**c**), and co-cultivation time (**d**) on oil palm transformation. “*” indicates statistically significant differences (*p* < 0.05); “**” indicates significant differences (*p* < 0.01); “***” indicates very significant differences (*p* < 0.001); “****” indicates extremely significant differences (*p* < 0.0001); ns indicates no significance.

To determine the optimal concentration of the *Agrobacterium* solution for oil palm transformation, we dipped the oil palm ECs into five different concentrations of *Agrobacterium*, corresponding to OD_600_ values of 0.3, 0.5, 0.6, 0.8, and 1.0. Following two to three cycles of glyphosate selection, the red fluorescent calli were observed and quantified, with the ratio serving as a criterion for evaluating transformation efficiency. Results indicated that the ratio of red fluorescent callus increased with *Agrobacterium* concentration, reaching 13.33% ±2.22% and 14.81% ± 1.28% (Figure 3a, Supplementary Table S3) at OD_600_ values of 0.5 and 0.6, respectively. Statistical analysis revealed no significant difference between these two concentrations. However, the ratio of red fluorescent callus declined significantly when *Agrobacterium* concentrations exceeded OD_600_ = 0.6, with partial browning observed at OD_600_ = 0.8 and worsened browning at OD_600_ = 1. Additionally, the *Agrobacterium* contamination ratio of callus was positively correlated with *Agrobacterium* concentration; no *Agrobacterium* contamination was observed at OD_600_ = 0.3, and the *Agrobacterium* contamination ratio remained relatively low at OD_600_ = 0.5 and 0.6. However, it increased dramatically at OD_600_ = 0.8 (Figure 3a, Supplementary Table S3). Based on these findings, we concluded that OD_600_ = 0.5 to 0.6 was optimal, as the fluorescence ratios for these two concentrations were significantly higher than those observed in the other test groups, while the *Agrobacterium* contamination ratios were acceptable.

To evaluate the effect of acetosyringone (AS) on transformation efficiency, oil palm ECs were immersed in *Agrobacterium* solutions with an optical density (OD_600_) of 0.512, containing AS concentrations of 0, 50 μM, 100 μM, 150 μM, 200 μM, and 250 μM. The calli were then placed on co-culture media corresponding to the AS concentrations and subsequently transferred to the glyphosate selection medium. After two to three cycles of glyphosate selection, the red fluorescent calli were observed and quantified. Results indicated that the red fluorescence ratio of callus at a 200 μM AS concentration was 16.30% ± 4.63%, significantly exceeding that of the other experimental groups. However, at an AS concentration of 250 μM, a decline in the number of red fluorescent callus was observed (Figure 3b, Supplementary Table S4). Thus, 200 μM was identified as the optimal AS concentration.

Regarding infection time, five intervals were tested: 0 min, 15 min, 30 min, 1 h, and 2 h. Oil palm ECs were immersed in an *Agrobacterium* solution with an OD_600_ of 0.548 containing 200 μM of AS. The results showed that the red fluorescence ratio of the callus was 14.07% ± 1.28% after 30 min of *Agrobacterium* inoculation, a ratio that was not significantly different from the 12.59% ± 1.28% observed after 1 h of infection. Both ratios, however, were significantly higher than those in the other experimental groups (Figure 3c, Supplementary Table S5). Based on these findings, the infection duration of 30 min to 1 h was recommended as optimal for *Agrobacterium* infection.

To determine the optimal duration for co-culture, four time intervals were evaluated: 24 h, 48 h, 72 h, and 96 h. The ECs were infected with an *Agrobacterium* solution at OD_600_ = 0.563 containing 200 μM of AS for 30 min. Results indicated that the highest red fluorescence ratio of 15.56% was achieved after 72 h of co-culture, significantly surpassing that of the other experimental groups (Figure 3d, Supplementary Table S6). Thus, 72 h was established as the optimal co-culture duration.

To identify the most effective antibiotics against *Agrobacterium*, Timentin and cefotaxime sodium were selected for evaluation. Twelve experimental groups were set up to investigate inhibition efficiency under the established optimal conditions. The results indicated that neither 400 mg/L of Timentin nor cefotaxime sodium effectively inhibited *Agrobacterium* growth, resulting in few red fluorescent calli. However, increasing the concentrations of both antibiotics to 500 mg/L significantly inhibited *Agrobacterium* proliferation, leading to an increased ratio of red fluorescent calli. Notably, the experimental groups A + 500 T and A + 500 Cs completely inhibited *Agrobacterium* proliferation, yielding red fluorescent callus ratios of 16.67% ± 2.62% and 12.96% ± 3.21%, respectively (Table 2, Supplementary Table S7). Although there was no significant difference in red fluorescent callus ratios between the A + 500 T and A + 500 Cs groups, the A + 500 T group exhibited a higher red fluorescent callus ratio than the other tested combinations, indicating it as the optimal antibacterial treatment.

### 2.4. Stable Transformation Using the TIPS-EiEPSPS/Glyphosate Selection System

**Figure 4:**
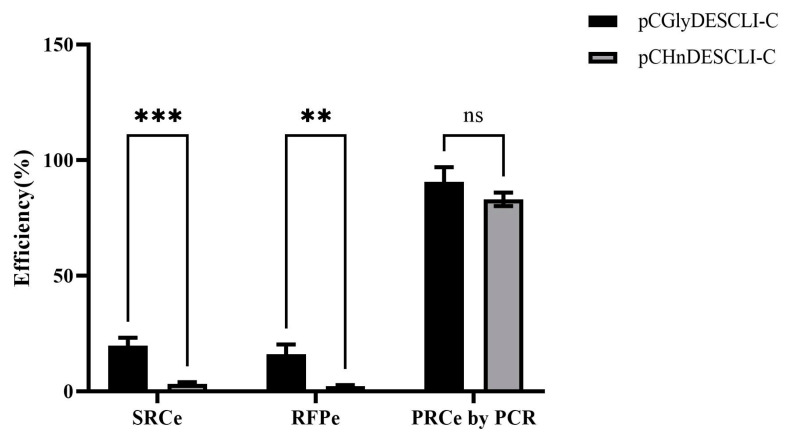
*Agrobacterium*-mediated transformation efficiency of two selection systems of oil palm. SRCe, suspected resistant calli efficiency; RFPe, the red fluorescence protein efficiency; and PRCe by PCR, positive calli efficiency of resistant calli by PCR. “**” indicates significant differences (*p* < 0.01), “***” indicates very significant differences (*p* < 0.001), ns indicates no significance.

Utilizing the optimal conditions established earlier, the novel *TIPS-EiEPSPS*/glyphosate selection system was implemented to generate transgenic materials from oil palm ECs as explants. Following four to five cycles of selection on a medium containing 3 mM of glyphosate, wild-type ECs exhibited progressive browning and ultimately died (Figure 2e). In contrast, the resistant calli derived from transgenic ECs displayed a light yellow coloration and vigorous growth, as indicated by the white arrows (Figure 2f), resembling the wild-type ECs (Figure 2c). Non-transgenic callus, however, demonstrated stunted growth and browning, failing to regenerate. The suspected glyphosate-resistant callus efficiency was 19.74% ± 3.44% across three replicates (Figure 4, Supplementary Table S8).

To compare the transformation efficiency of the *TIPS-EiEPSPS*/glyphosate selection system, we employed the *hpt*/hygromycin selection system to genetically transform the binary vector pCHnDESCLⅠ-C under identical conditions, except for the selection agent. After four to six cycles of selection on a medium containing 60 mg/L of hygromycin, the wild-type calli exhibited no further growth, experienced severe browning, and ultimately died (Figure 2g). Only a few transgenic calli were capable of generating new calli, and their proliferation rate was relatively slow. Most transgenic calli grown on the hygromycin selection medium showed significant browning, with minimal increase in volume (Figure 2h). Overall, the suspected hygromycin-resistant callus efficiency was 3.21% ± 0.74% (Figure 4, Supplementary Table S8), significantly lower than that observed with the *TIPS-EiEPSPS*/glyphosate selection system.

### 2.5. Molecular and RFP Analysis of Glyphosate-Resistant Callus in Oil Palm

**Figure 5:**
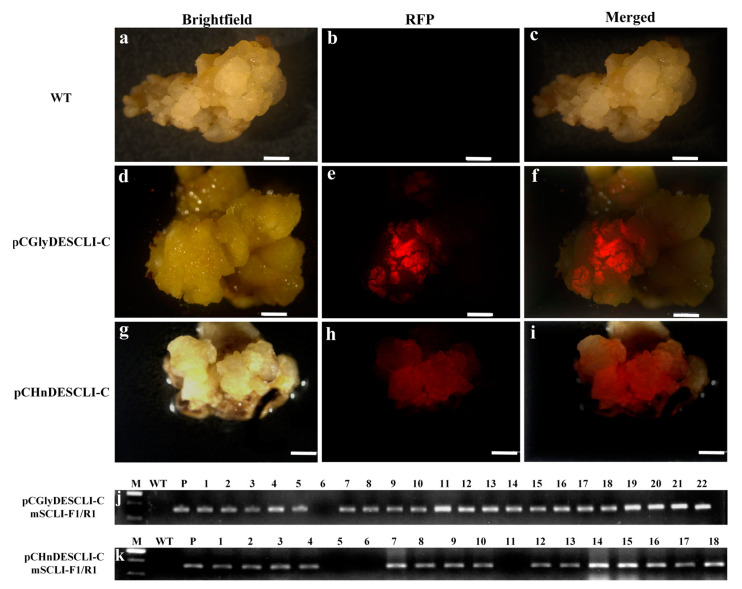
Visualization of RFP spots of glyphosate or hygromycin resistant calli and PCR detection results: (**a**–**c**), (**d**–**f**) and (**g**–**i**), visualization of brightfield, RFP and merged on wild-type ECs, glyphosate resistant ECs and hygromycin resistant ECs, respectively; (**j**,**k**), glyphosate resistance calli and hygromycin resistance calli detection by PCR (WT: wild-type EC; P, the vector pCGlyDESCLⅠ-C or pCHnDESCLⅠ-C plasmid used as the positive control, respectively; Lane 1–22, independent glyphosate-resistant calli samples; lane 1–18, independent hygromycin-resistant calli samples).

Red fluorescence visualization serves as an efficient and non-destructive method for initially selecting glyphosate-resistant calli prior to molecular analysis. Under green light excitation, scattered red dots appeared on the surface of the glyphosate-resistant callus, exhibiting bright red fluorescence (Figure 5e). In contrast, the wild-type callus did not display any red fluorescence (Figure 5b). Newly grown hygromycin-resistant callus also showed faint red fluorescence (Figure 5h). Preliminary results from the four-month-old resistant calli indicated the successful integration and expression of foreign genes within the oil palm genome. The ratio of red fluorescent callus selected using glyphosate was 16.00% ± 4.29%, significantly higher than the 2.30% ± 0.33% observed with *hpt*/hygromycin selection (Figure 4, Supplementary Table S8).

PCR analysis was conducted to identify the suspected resistant callus. The results indicated that most of the suspected resistant calli tested positive for the target gene. Among 22 randomly selected resistant callus samples, 21 exhibited clear and distinct amplification bands, classifying them as true positive transgenic calli; one sample failed to amplify the target band, resulting in a negative classification (Figure 5j). The estimated ratio of positive PCR results was 90.58% ± 6.33% (Figure 4, Supplementary Table S8). Most hygromycin-resistant calli also successfully amplified the correct band (Figure 5k), yielding a positive PCR ratio of 83.02% ± 2.87%, which was slightly lower than that of the glyphosate selection system, but without significant difference (Figure 4, Supplementary Table S8).

**Figure 6:**
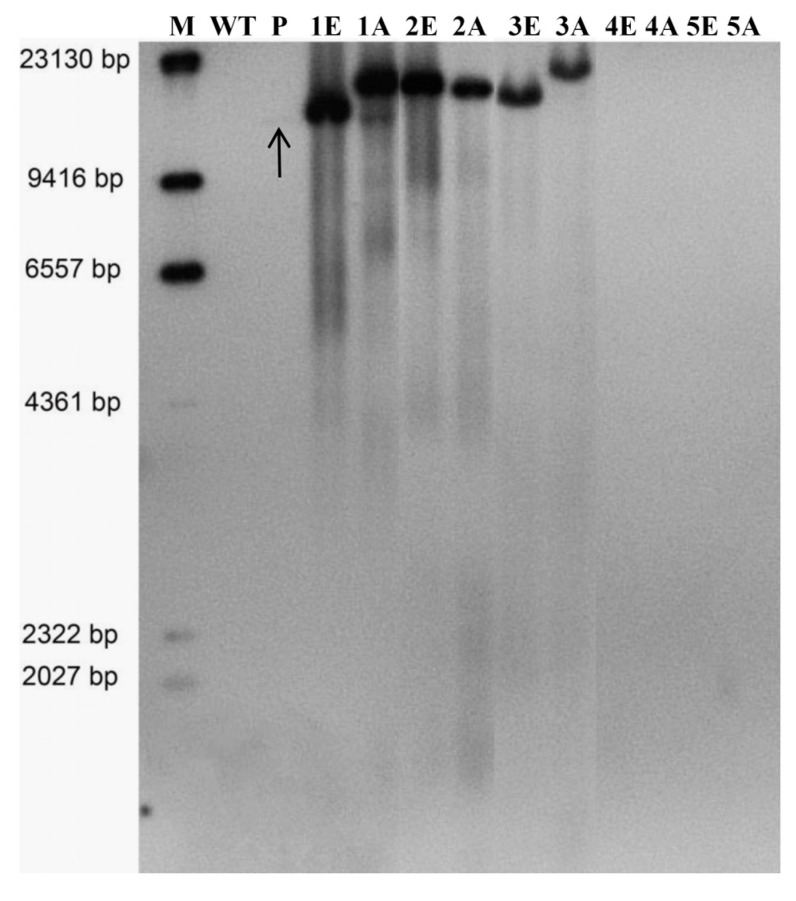
The positive transgenic and glyphosate resistance calli determined by Southern blot using the *EgmSCLⅠ* gene as a probe (EgmSCLI-Sb-F and EgmSCLI-Sb-R). WT, Wild-type EC of oil palm genomic DNA; P, The pCGlyDESCLⅠ-C vector plasmid used as the positive control, digested by *EcoRⅠ* (a fragment of about 13 kb was obtained), the arrow indicated the band; 1–5, Five PCR-positive ECs samples; E and A, EC samples genomic DNA digested by *EcoRⅠ* (a fragment larger than 5 kb) or *Avr Ⅱ* (a fragment larger than 11 kb), respectively.

To further verify the integration of foreign genes into the oil palm genome, Southern blot analysis was performed using a 556 bp probe amplified from the *EgmSCLⅠ* gene. Five glyphosate-resistant and PCR-positive calli samples were selected based on adequate sample weight. The genomic DNAs were digested with the restriction endonucleases *EcoR I* or *Avr II*; *EcoR I* cut at two sites and *Avr II* at one site on the pCGlyDESCLI-C vector (Figure 2a). The results showed that three samples produced clear hybridized bands of varying sizes, indicating different integration sites within the oil palm genome (Figure 6, Lane 1E-3A). Conversely, the other two samples displayed no bands, suggesting they were false-positive PCR results (Figure 6, Lane 4E-5A). Among the three positive samples, Sample 1 exhibited one hybridized band when digested with *EcoR I* (Figure 6, Lane 1E) and two bands when digested with *Avr II* (Figure 6, Lane 1A), suggesting that two copies may have integrated into the genome. The other two positive samples displayed only one hybridized band regardless of whether they were digested by *EcoR I* or *Avr II* (Figure 6, Lane 2E-2A), indicating a single copy integrated into the genomes of sample 2 and 3, respectively. Overall, the Southern blot results confirmed the successful creation of positive transgenic oil palm using the *TIPS-EiEPSPS*/glyphosate selection system, demonstrating impressive transformation efficiency.

## 3. Discussion

We previously developed a highly efficient *TIPS-EiEPSPS*/glyphosate selection system in rice, which resulted in transgenic rice exhibiting high levels of glyphosate resistance [26]. In this study, we successfully introduced this system into oil palm, marking the first report of a glyphosate selection system for this species. Historically, selection systems utilized in the genetic transformation of oil palm have included *bar*/Basta (glufosinate-ammonium, Bialaphos), *pmi*/mannose, *DOG-1*/2-DOG, and *hpt*/hygromycin. The *bar*/Basta selection system can lead to chimeras or transgenic escapes in the transgenic seedlings or plants [35]. While the *pmi*/mannose selection system has been employed to regenerate stable transgenic oil palm, it only inhibits the growth of non-transformed cells rather than eliminating them, necessitating extensive selection procedures to identify truly stably transformed plants [36,37]. Similarly, the *DOG-1*/2-DOG selection system is also a positive selection method, which can result in a high rate of regeneration of escapees [8,38]. The *hpt*/hygromycin selection system poses challenges in regenerating transgenic seedlings, with no reports to date on the successful acquisition of oil palm transgenic plants using this approach with oil palm EC as the receptor [39].

The *EPSPS*/glyphosate selection system has been effectively implemented in both monocotyledonous and dicotyledonous crops, including rice [21,26,40], wheat [18,28], corn [27], soybean [29,30,31], cotton [32], and *Brassica napus* [33]. The glyphosate concentration in the selection medium varies among different plant species. For instance, mature embryos of wheat transformed with the *CP4-EPSPS* and *GOX* genes were cultured on a selection medium containing 2 mM of glyphosate, leading to the generation of resistant callus [18]. Similarly, maize embryonic callus transformed with *Zm-EPSPS* and *GOX* genes were placed in a selection medium with 3 mM of glyphosate to obtain the resistant callus [27]. In rice, glyphosate concentrations of 1.2 mM and 2 mM were used for the transformation of *IvEPSPS* [21] and *G6-EPSPS* [40], respectively. In our previous study, we found that a concentration of 2.5–5 mM of glyphosate was more suitable for the transformation of *TIPS-EiEPSPS* in rice [26].

In the current study, we successfully applied this selection system to oil palm. Our findings indicate that a glyphosate concentration of 3 mM serves as the minimal inhibitory concentration for oil palm transformation. Furthermore, glyphosate concentrations exceeding 5 mM led to the significant inhibition of both transgenic and non-transgenic callus, which could substantially reduce transformation efficiency and prolong the selection cycle. Notably, compared to other selection agents, the browning of non-transgenic callus during glyphosate selection was less severe than that observed with hygromycin and Basta.

Transgenic research in oil palm commenced relatively late, with the earliest genetic transformation method being particle bombardment, which was tested by the Parveez team at the Malaysian Palm Oil Board (MPOB) in the early 1990s [6,41]. However, the transformation efficiency of particle bombardment was low, ranging from approximately 1.0% to 1.5% [42]. Nearly a decade later, *Agrobacterium*-mediated transformation was successfully applied to oil palm using EC as the explant and the *bar* gene as the selection marker. While PCR confirmed that resistant embryoid bodies and leaves contained the transgene, only 30% were identified as positive transgenic plants by Southern blot analysis, resulting in an overall transformation efficiency of just 0.7% [13]. Izawati et al. utilized *DOGR1* as the selection marker for transforming oil palm ECs via *Agrobacterium*-mediated transformation, achieving a PCR detection rate of 72% for resistant embryoids and a confirmation rate of 50% by Southern blot analysis, with a reported transformation efficiency of 1% [14]. Additionally, the *Agrobacterium*-mediated transformation of oil palm EC was achieved using the *hpt*/hygromycin selection system, with all resistant callus confirmed positive by PCR; however, no transformation efficiency data were provided [39].

Most studies lack a clear demonstration of selection efficiency due to missing data regarding the primary ECs and the resistant callus or embryoids. In our study using the *TIPS-EiEPSPS*/glyphosate selection system for oil palm, we achieved a suspected glyphosate-resistant callus efficiency of 19.74% ± 3.44%. The selection efficiency was estimated to be 17.88%. In contrast, the suspected hygromycin-resistant callus efficiency with the *hpt*/hygromycin selection system was only 3.21% ± 0.74%. The selection efficiency was estimated to be 2.66% (Supplementary Table S8). Experiments conducted under identical conditions demonstrated that the *TIPS-EiEPSPS*/glyphosate selection system outperformed the *hpt*/hygromycin system, indicating that glyphosate selection is more suitable for genetic transformation in oil palm. It is important to note that the sample size for Southern blot analysis was small due to the difficulty in obtaining the sufficient fresh weight of resistant callus. Therefore, future studies should involve more calli or regenerated plantlets for analysis.

Previous reports indicated that transgene escape is prevalent during the transition from transgenic callus to transgenic plants following selection [14,35]. Despite high positive PCR ratios for resistant embryoid bodies or callus, the regeneration of transgenic plants remained low [13,14,39], highlighting that the regeneration stage was the primary limitation in oil palm transgenic research. Reducing transgene escape is essential for enhancing transformation efficiency. One potential solution is to impose selection pressure during the regeneration stage to inhibit the development of non-transgenic plantlets. The *TIPS-EiEPSPS*/glyphosate selection system has been successfully validated for callus regeneration in rice [26], which may inform future work in oil palm. Additionally, the long regeneration cycle (at least 18 to 24 months) critically restricts functional research in oil palm. Even if glyphosate does not reduce transgene escape, the high ratio of positively resistant callus obtained from the *TIPS-EiEPSPS*/glyphosate selection system could serve as a rapid identification platform for gene function studies, particularly those related to fatty acid metabolism, since the fatty acid composition profile of oil palm fruit mesocarp can be effectively predicted by analyzing the fatty acid composition of oil palm ECs [43].

Currently, most genetically modified oil palm technologies are regulated by the Malaysian Palm Oil Board (MPOB), and available information is relatively limited. Consequently, it is imperative to leverage the experiences of previous researchers to explore and implement innovative methodologies, ultimately aiming to achieve genetically stable transgenic oil palm plants.

## 4. Materials and Methods

### 4.1. Plant Materials and Culture Medium

In this study, the embryonic calli (ECs) were induced from oil palm (*E. guineensis* ‘Tenera’) tender leaves and used for subsequent transgenic experiments [43]. Light yellow, granular, dry, and vigorous calli were selected and cultured in a subculture medium consisting of MS0 (Murashige and Skoog medium supplemented with glutamine, vitamin B1, ascorbic acid, sucrose, coconut water, agar, and activated carbon), along with an appropriate concentration of plant hormones. The callus was propagated through monthly subcultures. The cultures were maintained at a temperature of 28 ± 1 °C, with a light intensity of 3000 lx and a photoperiod of 12 h per day.

### 4.2. Codon Optimization and Vectors Construction

The red fluorescent protein gene *mscarlet-Ⅰ* (abbreviated as *mSCLⅠ*), derived from *mRed7*, was codon-optimized for oil palm (*Elaeis guineensis* Jacq.) using the OptimumGene tool (GenScript, Nanjing, China). The gene was driven by the seed-specific promoter Ltp2pro and enhanced by the CaMV35S promoter [44]. The optimized *EgmSCLI* expression cassette (GenBank No. PQ520475) was digested with *BamH Ⅰ* (Thermo, Waltham, MA, USA) and *Hind Ⅲ* (Thermo) and then ligated into the pCAMBIA1300 vector [45] (http://www.cambia.org/), preserving the restriction sites. This construct was designated as pCHnDESCLI.

The *EgmSCLⅠ* cassette, including cutting sites (*BsrG I*, *Stu I*, *Mlu I*, *Sma I*), was amplified from pCHnDESCLI-C and ligated into the basic vector pCEiEPSPS (GenBank No. MZ351488) [26] between *Afe I* (Thermo)and *Avr II* (Thermo), with *Avr II* retained, using a Lightening Cloning Kit (Gene-Foci, Beijing, China). This construct was designated as pCGlyDESCLI. Recombinant primers Gly-0-F and Gly-0-R (listed in Supplemental Supplementary Table S1) were used.

Additionally, the synthesized *com25* cassette was ligated into the pCHnDESCLI vector, which had been digested with *EcoR I* (Thermo)and *Kpn I* (Thermo)(with *Kpn I* retained), and the construct was designated pCHnDESCLI-C. The *com25* gene expression cassette, digested with *Stu I* (Thermo)and *Mlu I* (Thermo)from pCHnDESCLI-C, was then ligated into the pCGlyDESCLI vector, yielding the final construct pCGlyDESCLI-C with *Stu I* and *Mlu I* sites retained. The recombinant primers Gly-1-F and Gly-1-R (listed in Supplementary Table S1) were used. After verification by sequencing, the plasmids were transferred into *Agrobacterium tumefaciens* strain EHA105 for oil palm transformation.

### 4.3. Transformation of Oil Palm Embryonic Callus (EC)

Pre-culture: Embryogenic calli (ECs) were selected and transferred to the pre-culture medium (EgcPCM: MS0 + 200 μM acetosyringone (AS) + 100 mg/L cysteine) and incubated at 28 °C in the dark for 3–4 days.

Infection and co-culture: *Agrobacterium* was scraped and suspended in 50 mL of a MS0 liquid medium containing different concentrations of acetosyringone (0, 50, 100, 150, 200, 250 μM). The ECs were immersed in the bacterial suspension, adjusted to five optical densities at 600 nm (OD_600_ = 0.3, 0.5, 0.6, 0.8, 1.0), for various infection times (15 min, 30 min, 1 h, 2 h). After infection, the calli were dried on sterile filter paper in a sterile hood in the dark. The calli were then transferred to a co-culture medium (EgcCCM, same as EgcPCM).

Washing: The calli were co-cultured in an incubator at 19–22 °C in the dark for 24, 48, 72, or 96 h. After co-culturing, the calli were rinsed thoroughly with sterile water, then agitated in a solution containing antibiotics. After removing the liquid, the calli were dried on sterile filter paper, turning them periodically to ensure complete drying.

Selection: The selection medium (EgcSM: MS0 + 500 mg/L timentin + 3 mM glyphosate (Sigma-Aldrich, Saint Louis, MI, USA) or 60 mg/L hygromycin (Sigma-Aldrich, Saint Louis, MI, USA)) was prepared. The dried calli, previously soaked in *Agrobacterium* solution and washed, were transferred to the selection medium. The medium was refreshed every three weeks, and resistant calli were selected after 4–5 cycles of selection.

The determination of the minimum inhibitory concentration of glyphosate followed the same protocols, except for the *Agrobacterium* infection step. The suspected resistant callus efficiency (SRCe) was calculated as the number of newly grown resistant calli divided by the total number of calli. The red fluorescent callus ratio referred to the red fluorescence protein efficiency (RFPe) was calculated as the number of red fluorescent calli divided by the total number of calli. The positive callus verified by PCR (PRCe) was calculated as the number of PCR-positive calli divided by the number of newly grown resistant calli. The selection efficiency equals SRCe multiplied by PRCe.

A ratio of contamination referred to as the *Agrobacterium* contamination ratio is the number of *Agrobacterium*-contaminated calli divided by the total number of calli.

### 4.4. Observation of RFP Under Fluorescence Microscopy

Red fluorescent spots on the transgenic oil palm calli were observed using a Leica MZ10F stereomicroscope, with excitation at 569 nm and detection at 593 nm. Observations were made after 2 to 3 cycles of selection, and images of RFP-expressing cells were captured and analyzed using Leica Application Suite X (5.0.3.24880) software.

### 4.5. Genomic DNA Extraction and Polymerase Chain Reaction (PCR)

Genomic DNA was extracted from resistant callus that survived on a glyphosate-containing selection medium. To verify the presence of the *EgmSCLI* gene, primers mSCLI-F1 and mSCLI-R1 (listed in Supplementary Table S1) were used, amplifying a 412 bp fragment. PCR conditions included an initial denaturation at 94 °C for 3 min, followed by 30 cycles of 94 °C for 30 s, 65 °C for 30 s, 72 °C for 30 s, and a final extension at 72 °C for 5 min. PCR products were separated by electrophoresis on 1% (*w*/*v*) agarose gels.

### 4.6. Southern Blot Analysis

Southern blotting was used to detect the presence of transformed, PCR-positive EC [46]. The transformation vector pCGlyDESCLI-C served as the positive control, while total genomic DNA was extracted from freshly collected PCR-positive oil palm EC and wild-type EC. Southern blot analysis included the digestion of genomic and plasmid DNA, electrophoresis, capillary membrane transfer (Amersham Hybond-N+), hybridization and non-radioactive detection [47]. The target DNA fragment was detected using a probe, 556 bp fragment, which was amplified from the *EgmSCLⅠ* gene using primers EgmSCLI-Sb-F and EgmSCLI-Sb-R (listed in Supplementary Table S1).

### 4.7. Statistical Analysis

All samples were performed in triplicate to ensure reproducibility. Statistical analyses were performed using the software GraphPad Prism 9.0. The comparison method used ordinary one-way ANOVA and two-way ANOVA combined with Tukey’s multiple comparison test method to calculate the above significant differences. *p* < 0.05 indicates statistically significant differences (*), *p* < 0.01 indicates significant differences (**), *p* < 0.001 indicates very significant differences (***), and *p* < 0.0001 indicates extremely significant differences (****). α = 0.05.

## 5. Conclusions

In this study, we successfully established the *TIPS-EiEPSPS*/glyphosate selection system for oil palm using embryonic callus (EC) derived from tender leaves as explants, facilitated by *Agrobacterium*-mediated transformation. Comparative analyses of selection efficiency and the red fluorescence of resistant callus demonstrated that this system significantly outperformed the *hpt*/hygromycin selection system. This report represents the first application of the *TIPS-EiEPSPS*/glyphosate selection system in oil palm genetic transformation, offering a valuable alternative for advancing both basic research and the commercialization of transgenic oil palm.

## Figures and Tables

**Table 1:** Tests of the minimum inhibitory concentration of glyphosate and hygromycin on non-transgenic calli.

Medium + Selection Agents	Non-Transgenic Calli		
	No. of Calli Cultured	No. of Newly Grown Calli	Ratios of Newly Grown Calli
EgcSM	45	43	95.56% ± 3.85%
EgcSM + glyphosate 1 mM	39	13	33.33% ± 4.44%
EgcSM + glyphosate 3 mM	30	0	0.00%
EgcSM + glyphosate 5 mM	30	0	0.00%
EgcSM + glyphosate 10 mM	27	0	0.00%
EgcSM + glyphosate 20 mM	24	0	0.00%
EgcSM	27	27	100%
EgcSM + hygromycin 30 mg/L	27	13	48.15% ± 6.42%
EgcSM + hygromycin 60 mg/L	27	0	0.00%
EgcSM + hygromycin 90 mg/L	27	0	0.00%

EgcSM, oil palm EC selection medium.

**Table 2:** Effects of type and concentration of *Agrobacterium* antibiotics on oil palm RFP calli ratios.

Test Groups	Cleaning with Sterile Water	Cleaning with Antibiotic Solution	Antibiotics of Selection Medium	Proliferation of *A. tumefaciens*	Ratio of RFP Calli (%)
A + 400 Cs	Sterile Water	Sterile Water + 400 mg/L T	400 mg/L Cs	++	1.96 ± 3.40 bc
A + 400 T	Sterile Water	Sterile Water + 400 mg/L T	400 mg/L T	++	2.08 ± 3.61 bc
B + 400 Cs	Sterile Water + T + Tween	Sterile Water + 400 mg/L T	400 mg/L Cs	+++	0.00 ± 0.00 c
B + 400 T	Sterile Water + T + Tween	Sterile Water + 400 mg/L T	400 mg/L T	+++	0.00 ± 0.00 c
C + 400 Cs	Sterile Water	Sterile Water + 400 mg/L Cs	400 mg/L Cs	+++	0.00 ± 0.00 c
C + 400 T	Sterile Water	Sterile Water + 400 mg/L Cs	400 mg/L T	+++	1.85 ± 3.21 bc
A + 500 Cs	Sterile Water	Sterile Water + 500 mg/L T	500 mg/L Cs	−	12.96 ± 3.21 ab
A + 500 T	Sterile Water	Sterile Water + 500 mg/L T	500 mg/L T	−	16.67 ± 2.62 a
B + 500 Cs	Sterile Water + T + Tween	Sterile Water + 500 mg/L T	500 mg/L Cs	++	2.22 ± 3.85 bc
B + 500 T	Sterile Water + T + Tween	Sterile Water + 500 mg/L T	500 mg/L T	++	4.17 ± 3.61 bc
C + 500 Cs	Sterile Water	Sterile Water + 500 mg/L Cs	500 mg/L Cs	+	6.67 ± 2.31 bc
C + 500 T	Sterile Water	Sterile Water + 500 mg/L Cs	500 mg/L T	−	8.33 ± 2.89 b

The symbols “+” and “−” represent the relative comparison of the inhibition of *Agrobacterium* proliferation by antibiotics. The symbol “+” indicates a small number of proliferations of *A. tumefaciens;* “++” indicates a medium number of proliferations of *A. tumefaciens;* “+++” indicates a large number of proliferations of *A. tumefaciens*; while the symbol “−” denotes growth inhibition; In this context, T refers to Timentin, and Cs denotes Cefotaxime Sodium; The different letters (a, b, c) behind the numbers represent significant differences between groups. α = 0.05.

## Data Availability

All the data are included in this article.

## Supplementary Materials

The following supporting information can be downloaded at https://www.mdpi.com/article/10.3390/plants13233343/s1, Supplementary Table S1: List of primers used in the article; Supplementary Table S2: Effects of different concentration glyphosate and hygromycin on non-transgenic calli in selection; Supplementary Table S3: Effect of density of *A.tumefaciens* cell on oil palm RFP calli ratio; Supplementary Table S4: Effect of different concentration of acetosyringone(AS)on oil palm RFP calli ratio; Supplementary Table S5: Effect of different *A.tumefaciens* infection time on oil palm RFP calli ratio; Supplementary Table S6: Effect of different co-culture time on oil palm RFP calli ratio; Supplementary Table S7: Effect of type and concentration of *A.tumefaciens* antibioticson on oil palm RFP calli ratio; Supplementary Table S8: *Agrobacterium*-mediated transformation efficiency in the selection process of two selection systems of oil palm.
