# Supplementary material for: Highly Efficient Agrobacterium tumefaciens Mediated Transformation of Oil Palm Using an EPSPS-Glyphosate Selection System
Source: Plants (Basel). 2024 Nov 28;13(23):3343. doi: 10.3390/plants13233343 (PMC11644814; doi:10.3390/plants13233343)
Supplement: Supplementary file 1 [file plants-13-03343-s001.zip › Supplementary Materials-Plants-Highly efficient Agrobacterium tumefaciens mediated transformation of oil palm using EPSPS-Glyphosate selection system.pdf]

Supplementary Materials:

Table S1. List of primers used in the article.

Primer Name	Sequence（5'→3'）
Gly-0-F	5'-TCCTGCAGGCCCGGGCTGTACAAGGCCTACGCGTCCCGGGC GTATTGGCTAGAGCAGC-3'
Gly-0-R	5'-AAAATACGTACACGTGCCTAGGCTTATCTTTAATCATATTCC ATAGTC-3'
Gly-1-F	5'-AA CGGGCTGTACAAGGCCTGAGCTCGTGCAGCGTG-3'
Gly-1-R	5'-TACGCCCGGGACGCGTCCCGATCTAGTAACATAGATG-3'
mSCLI-F1	5'-ATCTCGCTGATTTCAAGACAACT-3'
mSCLI-R1	5'-TCCATAGTCCATACCATAGCACA-3'
EgmSCLI-Sb-F	5'-AAGGGGAGGGAAGGCCATAT-3'
EgmSCLI-Sb-R	5'-CCTGTAGAGTGCCGACCTTC-3'

Table S2. Effects of different concentration glyphosate and hygromycin on non-transgenic calli in selection.

Medium+selection agents	Non-transgenic calli		
	No.of calli cultured	No.of newly grown calli	Ratios of newly grown calli
EgcSM	15	15	100.00%
	15	14	93.33%
	15	14	93.33%
EgcSM+glyphosate 1mM	13	5	38.46%
	13	4	30.77%
	13	4	30.77%
EgcSM+glyphosate 3mM	10	0	0.00%
	10	0	0.00%
	10	0	0.00%
EgcSM+glyphosate 5mM	10	0	0.00%
	10	0	0.00%

	10	0	0.00%
	9	0	0.00%
EgcSM+glyphosate 10mM	9	0	0.00%
	9	0	0.00%
	8	0	0.00%
EgcSM+glyphosate 20mM	8	0	0.00%
	8	0	0.00%
	9	9	100.00%
EgcSM	9	9	100.00%
	9	9	100.00%
	9	4	44.44%
EgcSM+hygromycin 30mg/L	9	5	55.56%
	9	4	44.44%
	9	0	0.00%
EgcSM+hygromycin 60mg/L	9	0	0.00%
	9	0	0.00%
	9	0	0.00%
EgcSM+hygromycin 90mg/L	9	0	0.00%
	9	0	0.00%

[1] EgcSM,oil plam EC selection medium.

Table S3. Effect of density of *A.tumefaciens* cell on oil palm RFP calli ratio.

Density of *A.tumefaciens* cell （OD_{600}）	NO.of infected calli	NO.of RFP calli	Ratios of RFP calli	NO.of *A.tumefaciens* contaminated calli	Ratios of *A.tumefaciens* contamination
0.3	45	1	2.22%	0	0.00%
	45	2	4.44%	0	0.00%
	45	0	0.00%	0	0.00%
0.5	45	6	13.33%	1	2.22%
	45	7	15.56%	0	0.00%
	45	5	11.11%	0	0.00%
0.6	45	6	13.33%	2	4.44%
	45	7	15.56%	1	2.22%
	45	7	15.56%	1	2.22%
0.8	45	4	8.89%	13	28.89%
	45	4	8.89%	12	26.67%
	45	2	4.44%	14	31.11%
1.0	45	2	4.44%	21	46.67%
	45	3	6.67%	17	37.78%
	45	1	2.22%	20	44.44%

Table S4. Effect of different concentration of acetosyringone(AS)on oil palm RFP calli ratio.

AS concentration（μM）	NO.of infected calli	NO.of RFP calli	Ratios of RFP calli
0	45	1	2.22%
	45	0	0.00%
	45	0	0.00%
50	45	1	2.22%
	45	1	2.22%
	45	0	0.00%
100	45	0	0.00%

	45	2	4.44%
	45	2	4.44%
	45	3	6.67%
150	45	4	8.89%
	45	3	6.67%
	45	9	20.00%
200	45	8	17.78%
	45	5	11.11%
	45	1	2.22%
250	45	2	4.44%
	45	3	6.67%

Table S5. Effect of different *A.tumefaciens* infection time on oil palm RFP calli ratio.

Infection time	NO.of infected calli	NO.of RFP calli	Ratios of RFP calli
	45	0	0.00%
0min	45	0	0.00%
	45	0	0.00%
	45	3	6.67%
15min	45	4	8.89%
	45	3	6.67%
	45	6	13.33%
30min	45	7	15.56%
	45	6	13.33%
	45	6	13.33%
1h	45	6	13.33%
	45	5	11.11%
	45	1	2.22%
2h	45	2	4.44%
	45	1	2.22%

Table S6. Effect of different co-culture time on oil palm RFP calli ratio.

Co-culture time	NO.of infected calli	NO.of RFP calli	Ratios of RFP calli
	45	1	2.22%
24h	45	1	2.22%
	45	0	0.00%
	45	3	6.67%
48h	45	4	8.89%
	45	4	8.89%
	45	7	15.56%
72h	45	7	15.56%
	45	7	15.56%
	45	2	4.44%
96h	45	2	4.44%
	45	1	2.22%

Table S7. Effect of type and concentration of *A.tumefaciens* antibioticson on oil palm RFP calli ratio.

Test groups	NO.of infected calli	NO.of RFP calli	Proliferation of *A.tumefaciens*	Ratios of RFP calli
	17	1	+++	5.88%
A+400Cs	17	0	+++	0.00%
	17	0	+++	0.00%
	16	1	+++	6.25%
A+400T	16	0	+++	0.00%
	16	0	+++	0.00%
	21	0	+++	0.00%
B+400Cs	21	0	+++	0.00%
	21	0	+++	0.00%
B+400T	14	0	+++	0.00%

	14	0	+++	0.00%
	14	0	+++	0.00%
	15	0	+++	0.00%
C+400Cs	15	0	+++	0.00%
	15	0	+++	0.00%
	18	1	+++	5.56%
C+400T	18	0	+++	0.00%
	18	0	+++	0.00%
	18	2	-	11.11%
A+500Cs	18	2	-	11.11%
	18	3	-	16.67%
	22	4	-	18.18%
A+500T	22	3	-	13.64%
	22	4	-	18.18%
	15	1	++	6.67%
B+500Cs	15	0	++	0.00%
	15	0	++	0.00%
	16	1	++	6.25%
B+500T	16	0	++	0.00%
	16	1	++	6.25%
	25	1	+	4.00%
C+500Cs	25	2	+	8.00%
	25	2	+	8.00%
	20	1	-	5.00%
C+500T	20	2	-	10.00%
	20	2	-	10.00%

Table S8. *Agrobacterium*-mediated transformation efficiency in the selection process of two selection systems of oil palm.

Vector	Selective Agent	No.of calli inoculated	No.of suspected Resistant calli	Suspected resistant calli efficiency	No.of RFP calli	The red fluorescence efficiency	No.of Positive calli of resistant calli by PCR	Positive calli efficiency of resistant calli by PCR
pCGlyDESCLI-C	3mM glyphosate	225	36	16.00%	25	11.11%	31	86.11%
		225	46	20.44%	43	19.11%	45	97.83%
		180	41	22.78%	32	17.78%	36	87.80%
pCHnDESCLI-C	60mg/L hygromycin	154	6	3.90%	4	2.60%	5	83.33%
		206	5	2.43%	4	1.94%	4	80.00%
		211	7	3.32%	5	2.37%	6	85.71%
